# Localization of angiotensin converting enzyme in rabbit cornea and its role in controlling corneal angiogenesis in vivo

**Published:** 2010-04-23

**Authors:** Ajay Sharma, Daniel I. Bettis, John W. Cowden, Rajiv R. Mohan

**Affiliations:** 1 Harry S. Truman Memorial Veterans’ Hospital, Columbia, MO; 2 Mason Eye Institute, School of Medicine, University of Missouri-Columbia, MO; 3 Department of Ophthalmology, College of Veterinary Medicine, University of Missouri-Columbia, MO

## Abstract

**Purpose:**

The renin angiotensin system (RAS) has been shown to modulate vascular endothelial growth factor and angiogenesis. In this study we investigated (i) the existence of the RAS components angiotensin converting enzyme (ACE) and angiotensin II receptors (AT_1_ and AT_2_) in the rabbit cornea using in vitro and ex vivo models and (ii) the effect of enalapril, an ACE inhibitor, to inhibit angiogenesis in rabbit cornea in vivo.

**Methods:**

New Zealand White rabbits were used. Cultured corneal fibroblasts and corneal epithelial cells were used for RNA isolation and cDNA preparation using standard molecular biology techniques. PCR was performed to detect the presence of *ACE*, *AT_1_*, and *AT_2_* gene expression. A corneal micropocket assay to implant a vascular endothelial growth factor (VEGF) pellet in the rabbit cornea was used to induce corneal angiogenesis. Rabbits of the control group received sterile water, and the treated group received 3 mg/kg enalapril intramuscularly once daily for 14 days starting from day 1 of pellet implantation. The clinical eye examination was performed by slit-lamp biomicroscopy. We monitored the level of corneal angiogenesis in live animals by stereomicroscopy at days 4, 9, and 14 after VEGF pellet implantation. Collagen type IV and lectin immunohistochemistry and fluorescent microscopy were used to measure corneal angiogenesis in tissue sections of control and enalapril-treated corneas of the rabbits. Image J software was used to quantify corneal angiogenesis in the rabbit eye in situ.

**Results:**

Our data demonstrated the presence of *ACE*, *AT_1_*, and *AT_2_* expression in corneal fibroblasts. Cells of corneal epithelium expressed *AT_1_* and *AT_2_* but did not show *ACE* expression. Slit-lamp examination did not show any significant difference between the degree of edema or cellular infiltration between the corneas of control and enalapril-treated rabbits. VEGF pellet implantation caused corneal angiogenesis in the eyes of vehicle-treated control rabbits, and the mean area of corneal neovascularization was 1.8, 2.8, and 3.2 mm^2^ on days 4, 9, and 14, respectively. Enalapril treatment caused a notable decrease in corneal neovascularization of 44% (1 mm^2^), 28% (2.1 mm^2^), and 31% (2.2 mm^2^) on the three tested time points, respectively. The immunostaining of corneal tissue sections with collagen type IV and lectin confirmed the presence of blood vessels, with enalapril-treated rabbit corneas showing a lesser degree of blood vessel staining.

**Conclusions:**

Corneal cells show expression of tissue RAS components, such as *ACE*, *AT_1_*, and *AT_2_*. Treatment with ACE inhibitor enalapril markedly decreased corneal angiogenesis in a rabbit model of VEGF-induced corneal neovascularization, suggesting that ACE inhibitors may represent a novel therapeutic strategy to treat corneal angiogenesis.

## Introduction

Corneal clarity is necessary for normal vision. Corneal insult, such as trauma, chemical injury, infections, or immune disorders, can lead to corneal neovascularization and loss of corneal transparency [1,2]. Clinical management of corneal neovascularization is challenging and preexisting neovascularization in acceptor eye also significantly increases the risk of corneal transplant rejection [3]. A variety of agents, such as corticosteroids [4], cyclosporine [5], indomethacin [6], methotrexate [7], rapamycin [8], low-molecular weight heparin sulfate [9], and thalidomide [10], have been evaluated in animal models of angiogenesis to treat corneal neovascularization but have shown limited success. Bevacizumab, an anti-vascular endothelial growth factor (VEGF) antibody, has recently been successfully tested in patients of corneal neovascularization [11]. Although bevacizumab is well tolerated, multiple applications may be required for effective treatment, and this can be expensive. Therefore, there is an unmet medical need for clinically effective agents that can inhibit corneal neovascularization.

The renin angiotensin system (RAS) is among the most powerful regulators of blood pressure and plasma volume [12]. The RAS consists of kidney renin (converts circulating plasma protein angiotensinogen into angiotensin I) and angiotensin-converting enzyme (ACE) that converts angiotensin I to angiotensin II (Ang II). Besides the circulating RAS, the presence of a tissue-specific local RAS is also well documented for brain, heart, pancreas, kidney, blood vessels, and gonads [12]. In ocular tissue, multiple studies have tested the presence of the tissue RAS, demonstrating mRNA and protein expression of renin, angiotensinogen, and Ang II receptors in ciliary bodies [13,14], choroid [15], ganglion cells [14], pigmented epithelial cells [16,17], and Müller cells of the retina [18]. The presence of angiotensin and ACE enzyme activity have also been detected in the vitreous fluid [19]. Among retina cells, the presence of renin has been shown in pigmented epithelial cells but not in neural retina, whereas angiotensinogen and ACE could be detected in both pigmented epithelial cells and neural retina [16]. These findings suggest that RAS expression is not constant, even in the various cell types of a single tissue such as retina. A few researchers have tested the presence of the RAS in cornea, but these, performed in rodent, dog, and primate eyes, have shown contradictory findings [14,15,17]. Savaskan et al. [14] and Usui et al. [20] reported expression of ACE and Ang II receptors (AT_1_) in the primate and mouse cornea, whereas Sheota et al. [15] and Murata et al. [17] did not detect ACE or AT_1_ in dog and rat cornea. The detailed investigation of RAS components in different corneal cell types is still missing.

Typically, Ang II is implicated in controlling plasma volume, electrolyte balance, and blood pressure [12]. Recently, Ang II has been ascribed to modulate many more pro-angiogenic activities, including the proliferation and chemotaxis of vascular smooth muscle and endothelial cells and increased transcription of VEGF [21-24]. These reports suggest that Ang II may have a role in promoting angiogenesis. Thus, it is reasonable to speculate that suppression of Ang II can inhibit angiogenesis. Numerous studies have demonstrated that ACE inhibitors (known to block Ang II formation) decrease cellular proliferation, angiogenesis, and VEGF expression in different tumor cell lines [25-27]. Furthermore, the anti-angiogenic effect of ACE inhibitors has been shown in various experimental animal models of cancer. In these studies, ACE inhibitors showed a decrease in tumor growth and VEGF levels [25-30]. In the eye, ACE inhibitors have shown a significant decrease in choroidal [31] and retinal vascularization [32] in rodent models in vivo. A recent clinical trial performed with an ACE inhibitor, enalapril, showed 65% reduction in progression of diabetic retinopathy in enalapril-treated patients [33]. We hypothesized that inhibition of RAS activity with ACE inhibitor can prevent angiogenesis in rabbit cornea in vivo. Therefore, we evaluated the effect of enalapril on corneal neovascularization using a well-defined VEGF-induced rabbit model of corneal neovascularization. The expression of ACE enzyme and the angiotensin receptors AT_1_ and AT_2_ in rabbit corneal epithelium and fibroblasts was also investigated.

## Methods

### Rabbits and corneal cultures

Female New Zealand White rabbits (Myrtle laboratories Inc., Thompson’s Station, TN) weighing 2.5–3.0 kg were used in this study. The study was approved by the Institutional Animal Care and Use Committee (Harry S. Truman Memorial Veterans’ Hospital, Columbia, MO). Animals were treated in accordance with the tenets of the ARVO Statement for the Use of Animals in Ophthalmic and Vision Research. The cells of corneal epithelium were collected by scraping the epithelium with a surgical blade. Primary corneal fibroblasts were generated from the rabbit corneas as previously described [34]. Briefly, cornea was washed with cell culture medium, and epithelium and endothelium were removed by gentle scraping with a scalpel blade. Corneal stroma was cut into small pieces and incubated in a humidified (90%) CO_2_ incubator at 37 °C in Dulbecco’s modified Eagle’s medium, containing 10% fetal bovine serum, to obtain rabbit corneal fibroblasts. Seventy percent confluent cultures of rabbit corneal fibroblasts were used for experiments.

### RNA extraction, cDNA synthesis, and PCR

Total RNA from the cells was extracted using the RNeasy kit (Qiagen Inc., Valencia, CA) and was reverse-transcribed to cDNA, following the vendor’s instructions (Promega, Madison, WI). Briefly, cells were lysed in 350 µl RLT buffer (Qiagen Inc., Valencia, CA) containing β-mercaptoethanol, followed by equal volume of ethanol addition. The solution was then applied onto RNeasy column and RNA was eluted from the column using 30 µl of RNAse free water. The eluted RNA was reverse transcribed to cDNA using oligo(dT) primers, AMV reverse transcriptase (Promega), reverse transcriptase buffer, dNTPs, RNasin. The reverse transcription reaction was carried out at 42 °C for 30 min followed by enzyme inactivation by heating at 90 °C for 2 min. PCR was performed for the detection of *ACE*, *AT_1_*, and *AT_2_*. A 50-µl reaction mixture containing 3 µl cDNA, 2 µl forward (200 nM) and 2 µl reverse (200 nM) primer, 3.125 mM of deoxynucleotide triphosphates (dNTPs) and Taq polymearse was run one cycle at 95 °C for 3 min, then 40 cycles of 95 °C 30 s, followed by 55 °C 30 s and 55 °C for 60 s, using a thermocycler (Bio-Rad Laboratories, Hercules, CA). For *ACE*, the forward primer sequence 5′-ACG AGC ACG ACA TCA ACT TCC TCA-3′ and reverse primer sequence 5′-AGT AGT TCA TCA TGG CCG AGG CT-3′ were used. For *AT_1_*, the forward primer sequence was 5′-AGG ATG ACT GTC CCA AAG CTG GAA-3′, and the reverse primer sequence was 5′-ACG TTT CGG TGG ATG ATA GCT GGT-3′. For *AT_2_*, the forward primer sequence was 5′-TGA GAA ATA TGC CCA GTG GTC GGT-3′, and the reverse primer sequence was 5′-ATA ATC CAG ATG GGC CTC AAG CCA-3′. β-actin (*ACTB*) was used as a positive control gene: forward primer (5′-AGG CCA ACC GCG AGA AGA TGA CC-3′), reverse primer (5′-GAA GTC CAG GGC GAC GTA GCA C-3′). cDNA samples were prepared from two different rabbits. Each PCR was repeated at least three times.

### Corneal angiogenesis in rabbits

Eight female New Zealand White rabbits (2.5–3.0 kg) were divided in two groups. Animals were anesthetized by intramuscular injection of ketamine hydrochloride (50 mg/kg) and xylazine hydrochloride (10 mg/kg). In addition, topical proparacaine hydrochloride 0.5% (Alcon, Ft. Worth, TX) was applied to each eye just before surgery. A corneal micropocket assay was performed in the rabbit eyes under general and local anesthesia. Only one eye of each animal was used for the surgical procedure, and the contralateral eye served as a naive control. A wire speculum was positioned in the eye, and a sucralfate-hydron pellet containing 650 ng of VEGF (PeproTech, Rocky Hill, NJ) was implanted in the cornea micropocket. Rabbits of the control group received sterile water, and the treated group received 3 mg/kg enalapril (Sigma Aldrich Inc., St. Louis, MO) via intramuscular injection once daily for 14 days starting from day 1 of pellet implantation.

### Stereomicroscopy and slit-lamp examination

The level of neovascularization in the cornea was monitored with a micrometer-calibrated stereomicroscope (Leica, Wetzlar, Germany) equipped with a digital camera (SpotCam RT KE; Diagnostic Instruments Inc., Sterling Heights, MI). Rabbit eyes were imaged at 4, 9, and 14 days after pellet implantation for quantitative analysis of corneal neovascularization. Slit-lamp biomicroscopy was also performed on naive and VEGF-implanted untreated and enalapril-treated rabbit eyes. Qualitative clinical evaluation data about the redness, edema, and inflammation were recorded.

### Tissue collection

Rabbits were humanely euthanized with pentobarbitone (150 mg/kg) under general anesthesia. Corneas were removed with forceps and sharp Westcott scissors, embedded in liquid optimal temperature cutting compound (OCT) compound (Sakura FineTek, Torrance, CA) within a 24 mm×24 mm×5 mm mold (Fisher, Pittsburgh, PA), and snap frozen. The frozen tissue blocks were maintained at −80 °C. Tissue sections (7 µm) were cut with a cryostat (HM 525M; Microm GmbH, Walldorf, Germany). Sections were placed on 25 mm×75 mm×1 mm microscope Superfrost Plus slides (Fisher), and maintained frozen at −80 °C until staining.

### Immunostaining for collagen type IV and lectin

Collagen type IV is a major component of the basal lamina of blood vessels. We therefore performed collagen type IV immunohistochemistry to detect the presence of blood vessels. Blood vessel staining was also confirmed with lectin (obtained from *Lycopersicon esculatum*). Briefly, the tissue sections were equilibrated at room temperature to thaw OCT. Tissue sections were washed with 1× HEPES buffer for 15 min, blocked with 5% bovine serum albumin for 30 min, and incubated with 1:50 dilution (in 1× HEPES buffer) of goat polyclonal antibody for collagen type IV (catalog no. sc9302; Santa Cruz Biotechnology Inc., Santa Cruz, CA) for 90 min. Donkey antigoat Alexa 594 secondary antibodies (catalog no. A-11058; Invitrogen, Carlsbad, CA) were used at a dilution (in 1× HEPES buffer) of 1:500 for 1 h. For lectin staining, sections were incubated with a solution of 20 µg/ml Texas red-conjugated tomato lectin (Vector laboratories, Burlingame, CA) for 30 min. The sections were washed three times in HEPES buffer, mounted in vectashield containing 4'-6-diamidino-2-phenylindole (DAPI; Vector laboratories), and viewed and photographed with a Leica fluorescent microscope (Leica DM 4000B; Leica) equipped with a digital camera (SpotCam RT KE).

### Quantification of corneal neovascularization

Adobe Photoshop CS2 (Adobe systems, San Jose, CA) and National Institutes of Health Image J 1.38X (NIH, Bethesda, MD) software were used for quantifying corneal neovascularization. The quantification of corneal vasculature in the eye of the live rabbit at different selected days was performed with stereo and slit-lamp microscopy. Images of the eye covering corneal neovascularization were taken with a digital camera fitted in a microscope for quantification analysis.

### Statistical analysis

The results were expressed as mean±standard error of the mean (SEM). Statistical analysis was performed using two-way analysis of variance (ANOVA) followed by Bonferroni multiple comparisons test. A value of p<0.05 was considered statistically significant.

## Results

### Detection of *ACE*, *AT_1_*, and *AT_2_* in rabbit corneal epithelium and fibroblasts

**Figure 1:**
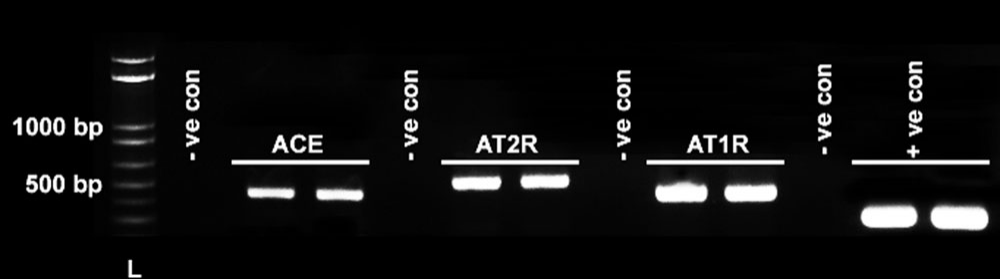
Representative image showing detection of *ACE*, *AT_1_*, and *AT_2_* mRNA expression in rabbit corneal fibroblasts with PCR. Appropriate size amplification products for *ACE* (469 bp), *AT_1_* (463 bp), and *AT_2_* (551 bp) were detected in two independent cDNA samples of corneal fibroblast prepared from different rabbits. -ve con denotes negative controls that contained *ACE*, *AT_1_*, or *AT_2_* primers but water instead of cDNA. + ve con represents the positive control of β-actin (*ACTB*; 350 bp). L denotes 1 kb plus the DNA ladder.

Figure 1 shows the presence of *ACE*, *AT_1_*, and *AT_2_* in rabbit corneal fibroblasts. An appropriate size amplification product of 469 bp was detected for *ACE* in two independent rabbit corneal fibroblast samples. Amplification products for *AT_1_* and *AT_2_*, corresponding to the product size of 463 bp and 551 bp, respectively, were also present in the rabbit corneal fibroblast cDNA samples. No PCR product was detected in the negative controls that contained primers for *ACE*, *AT_1_*, or *AT_2_* but no cDNA. *ACTB* was used as a positive control. The appropriate size amplification product of *ACTB* at 350 bp was detected in all PCRs (Figure 1).

**Figure 2:**
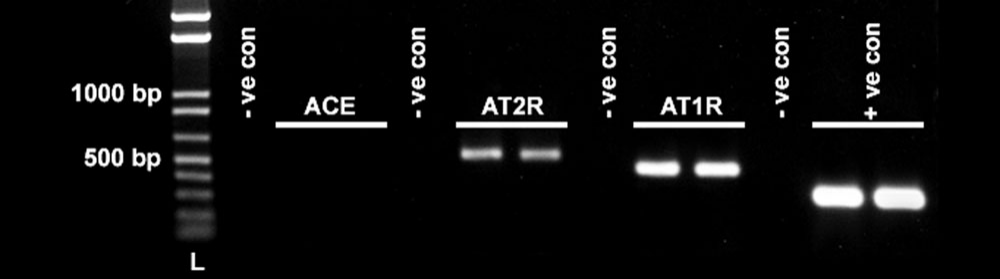
Representative image showing *AT_1_* and *AT_2_* mRNA expression in rabbit corneal epithelium detected with PCR. Appropriate size amplification products for *AT_1_* (463 bp) and *AT_2_* (551 bp) were detected in two independent rabbit corneal epithelium cDNA samples prepared from different rabbits. No signal for ACE was detected. -ve con denotes negative controls that contained *ACE*, *AT_1_*, or *AT_2_* primers but water instead of cDNA. + ve con represents the positive control of β-actin (*ACTB*; 350 bp). L denotes 1 kb plus the DNA ladder.

Figure 2 shows the presence of *AT_1_* and *AT_2_* in the epithelial cells of rabbit cornea. Anticipated size PCR products of 463 bp and 551 bp for *AT_1_* and *AT_2_*, respectively, were detected in the two independent cDNA samples of rabbit epithelium. On the other hand, no amplification product for *ACE* was detected, suggesting that rabbit epithelium does not express ACE enzyme. Negative controls containing appropriate primers and water instead of cDNA samples were also analyzed and did not show any amplification products. *ACTB* was used as a positive control and showed a PCR product band of 350 bp (Figure 2).

### Slit-lamp examination of rabbit eyes

**Figure 3:**
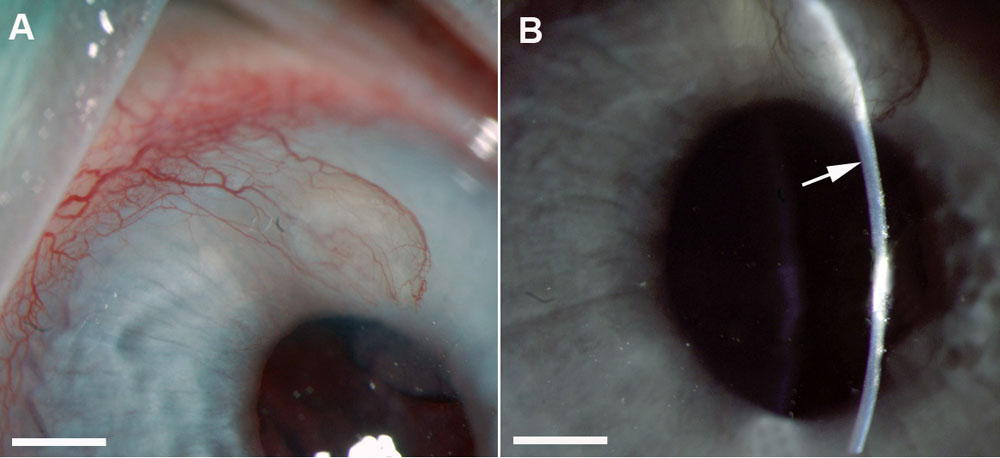
Representative broad-beam (**A**) and narrow-beam (**B**) slit-lamp biomicroscopy images of VEGF-implanted rabbit corneas. Corneas did not show any cellular infiltrate or edema in the area inferior to VEGF pellet (as shown by arrow). A mild level of edema is noticeable around the area of VEGF pellet. The scale bar denotes 2 mm.

Figure 3 shows broad beam and narrow beam slit-lamp pictures of the eyes of control and enalapril-treated rabbits. A qualitative comparison between the control and enalapril-treated corneas did not reveal any significant difference in the degree of redness, edema, or cellular infiltration.

### Effect of enalapril on corneal angiogenesis

**Figure 4:**
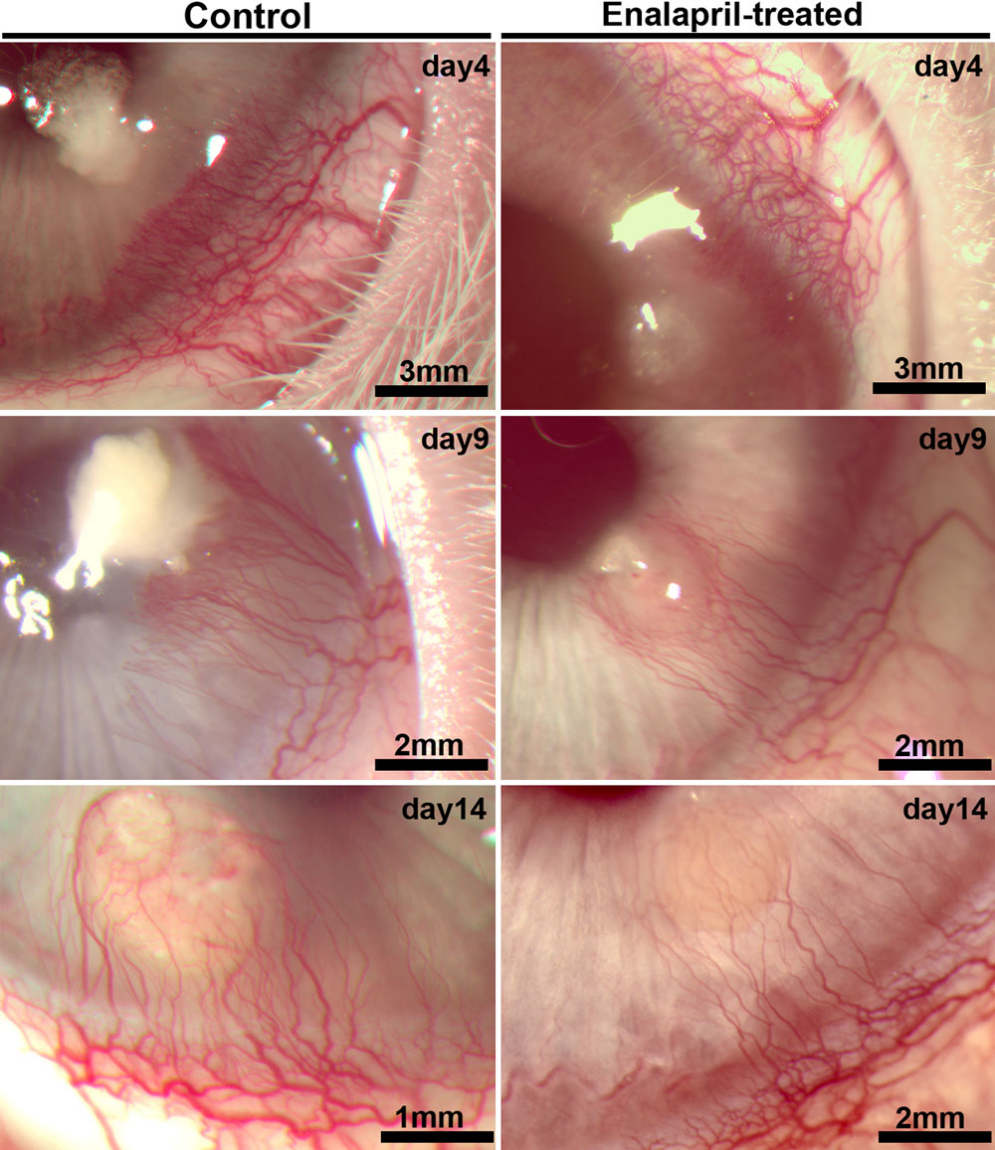
Representative stereomicroscopy images showing VEGF-induced neovascularization in the cornea of control and enalapril-treated rabbit eyes. The rabbit eyes were imaged on day 4, 9, and 14 days after VEGF implantation to monitor corneal angiogenesis in control and enalapril treated rabbits.

Figure 4 shows stereomicroscopic images depicting the localization and extent of corneal neovascularization in control and enalapril-treated rabbit eyes. VEGF pellet implantation produced corneal neovascularization in both control and enalapril-treated rabbits. The images captured on day 4, 9, and 14 show a time-dependent increase in corneal blood vessels in both the control and enalapril-treated groups. However, a notable decrease in corneal neovascularization can be seen in eyes of rabbits treated with enalapril (Figure 4).

**Figure 5:**
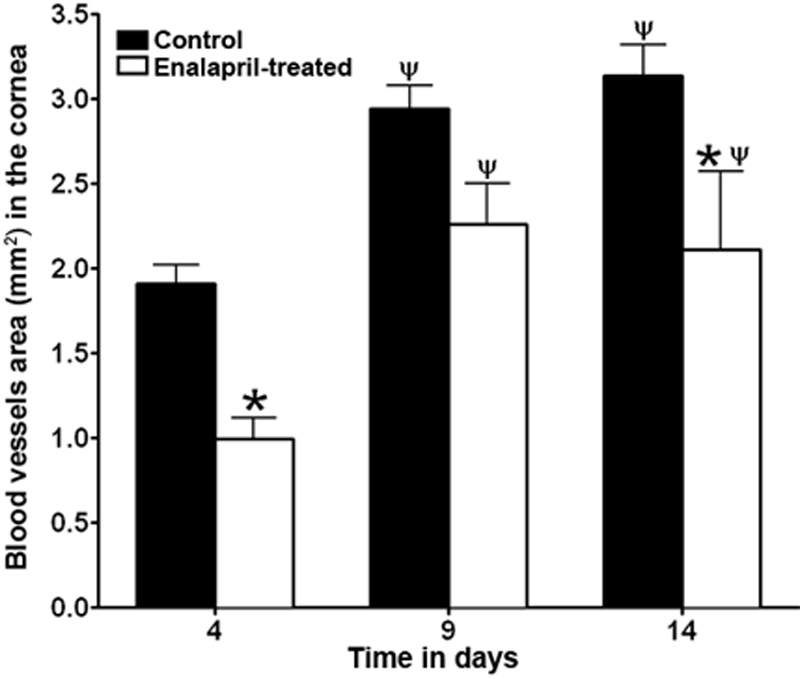
Digital quantification of VEGF-induced corneal neovascularization in enalapril-treated (white bar) and enalapril-untreated control (black bar) eyes of live rabbit performed on days 4, 9, and 14. ψ denotes a p<0.01 and shows a significant value of corneal neovascularization on day 9 and 14 compared to day 4; * denotes a p<0.05 and shows a significant value of corneal neovascularization in enalapril-treated rabbit compared to enalapril-untreated controls. Bars represent standard error.

For quantitative comparisons between control and treated animals, we calculated the area of corneal neovascularization using Image J software. Figure 5 shows the mean area of corneal neovascularization in control and enalapril-treated rabbits at the three tested time points. Control corneas showed a mean corneal neovascularization area of 1.8 mm^2^ at day 4. The area of corneal neovascularization noted at day 9 and day 14 was 2.8 and 3.2 mm^2^, respectively, and it was significantly higher compared to day 5 (p<0.01; shown by ψ in Figure 5). Corneas of enalapril-treated rabbits showed a mean corneal neovascularization area of 1 mm^2^ on day 4, which increased significantly (p<0.01; shown by ψ in Figure 5) to 2.1–2.2 mm^2^ on day 9 or day 14. A relative comparison between control and enalapril-treated rabbits revealed a mean decrease in corneal neovascularization of 44%, 28%, and 31% on day 4, 9, and 14, respectively, and the decrease was statistically significant at day 4 and day 14 (p<0.01; shown by the asterisk in Figure 5).

**Figure 6:**
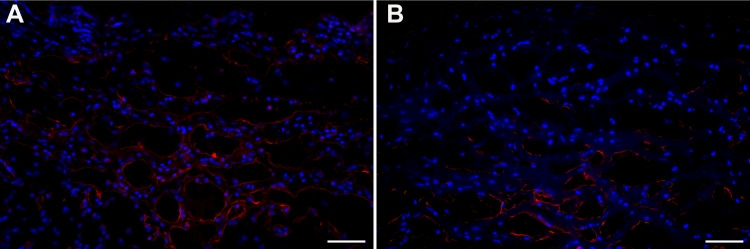
Representative immunohistochemistry image showing collagen type IV staining of blood vessels (red staining) in tissue sections of rabbit corneas collected 14 days after sterile water (**A**) or enalapril treatment (**B**). The images show angiogenesis in the central cornea region. Enalapril-treated corneas showed fewer blood vessels. Nuclei are stained blue with DAPI. The scale bar denotes 100 µm.

The effect of enalapril on corneal angiogenesis was further confirmed by collagen type IV and lectin staining of the blood vessels in the corneal tissue sections of control and enalapril-treated rabbits. Figure 6 demonstrates the localization and level of collagen type IV-stained blood vessels in corneal tissue sections of control and enalapril-treated rabbits. Collagen type IV and lectin staining (data not shown) revealed fewer blood vessels in corneal sections obtained from enalapril-treated rabbits compared to control corneal sections (Figure 6).

## Discussion

The presence of the RAS in a wide variety of nonocular tissues, including brain, heart, pancreas, and gonads, has been reported [12]. In the eyes, renin, angiotensin, and angiotensinogen protein and mRNA expression have been reported in retina [14,16,17], choriod [15], and ciliary bodies [13,14] but were not tested in various cells of the cornea [13-17]. Among various cell types of retina, renin expression has been detected in pigmented epithelial cells but not in neural retina, whereas angiotensinogen and ACE expression were demonstrated in both pigmented epithelial cells and neural retinal cells [13]. Similarly, expression of AT_1_ has been mainly reported in retinal ganglion and photoreceptor cells but not in pigmented epithelial cells [14,15,17]. These studies suggest that expression of RAS components in different cells of a tissue may vary and may possess unique cellular distributions. Our study demonstrates that both corneal epithelium and fibroblasts express *AT_1_* and *AT_2_*, whereas *ACE* is expressed in corneal fibroblasts but not in corneal epithelium. Our data are in agreement with earlier studies that detected the presence of AT_1_ and ACE in whole corneal homogenates of the mouse and human cornea [14,20]. It is also evident from the literature that corneal RAS expression varies by species, as the presence of AT_1_ and ACE was not detected in rat [17] and dog [15] cornea, respectively. To the best of our knowledge, this is the first study to report cellular localization of ACE, AT_1_, and AT_2_ expression in two different cells of the rabbit cornea.

Transdifferentiation of fibroblast to myofibroblast is thought to be a central event in the pathogenesis of corneal fibrosis. In nonocular tissues, myofibroblasts are shown to express high levels of ACE that can generate large amounts of Ang II [35]. The elevated level of Ang II is reported to modulate expression of collagen, fibronectin, and many other extracellular matrix proteins, suggesting that the RAS expressed locally in fibroblasts may participate in fibrosis and wound healing [36-38]. Elevated levels of angiotensin receptors were reported in skin fibroblasts during wound healing, which further supports this notion [39]. Based on these studies in nonocular tissues, we speculate that the presence of corneal RAS components demonstrated in our study may play a role in corneal wound healing and fibrosis. Future studies will investigate the role of the RAS in corneal wound healing and fibrosis. Besides fibrosis, the corneal RAS may participate in regulation of the inflammatory response, as suggested by a recent study demonstrating a marked increase in *ACE*, *AT_1_*, and angiotensinogen gene expression in a mouse corneal model of a nylon suture-induced corneal injury [20].

Ang II is the biologically active end product of the RAS. Accumulating evidence suggests that Ang II promotes endothelial and vascular smooth muscle cell proliferation and chemotaxis besides increasing VEGF expression [21-24]. Further support for the pro-angiogenic effect of Ang II comes from in vivo studies that showed Ang II implantation induces blood vessel formation in rabbit cornea and in the chick chorio-allantoic membrane. These investigations provide strong support about the role of Ang II in angiogenesis [40,41]. Most importantly, these observations led to a novel interventional strategy to control angiogenesis by blocking Ang II formation. ACE inhibitors are known to block Ang II formation, and numerous studies have demonstrated that ACE inhibitors decrease cellular proliferation, VEGF expression, and inhibit angiogenesis in culture and in animal models of cancer [25-30]. These studies and our data showing expression of the RAS locally in the cornea led us to hypothesize that ACE inhibitors can inhibit corneal neovascularization in rabbit eye in vivo. We tested this hypothesis using a well-defined rabbit model of corneal neovascularization, and our results showed that enalapril treatment significantly prevents VEGF-induced corneal neovascularization in rabbit eye in vivo. A relatively low dose (3 mg/kg) of enalapril was used in our experiments to avoid the potential risk of hypotension [42]. However, it is likely that higher or multiple doses of enalapril may show a greater anti-angiogenic effect in the cornea. The intramuscular administration of enalapril over topical application was selected based on pharmacokinetic information available in the literature.

In conclusion, this study demonstrates the presence of AT_1_ and AT_2_ in rabbit corneal fibroblasts and epithelial cells and ACE expression in corneal fibroblasts of rabbit cornea. Furthermore, we found a significant decrease in VEGF-induced corneal neovascularization in rabbit in vivo with enalapril, an ACE inhibitor. ACE inhibitors represent a novel therapeutic strategy to treat corneal neovascularization; however, this requires further investigation.
